# Extraordinarily Adaptive Properties of the Genetically Encoded Amino Acids

**DOI:** 10.1038/srep09414

**Published:** 2015-03-24

**Authors:** Melissa Ilardo, Markus Meringer, Stephen Freeland, Bakhtiyor Rasulev, H. James Cleaves II

**Affiliations:** 1 Centre for GeoGenetics, Natural History Museum, University of Copenhagen, Øster Voldgade 5–7, 1350 Copenhagen K, Denmark; 2 University of Hawaii at Manoa, 2500 Campus Rd, Honolulu, HI 96822, USA; 3 German Aerospace Center (DLR), Earth Observation Center (EOC), Münchner Straße 20, 82234 Oberpfaffenhofen-Wessling, Germany; 4 University of Maryland Baltimore County, 1000 Hilltop Cir, Baltimore, MD 21250, USA; 5 Interdisciplinary Center for Nanotoxicity, Department of Chemistry and Biochemistry, Jackson State University, 1400 J.R. Lynch St. Jackson, MS, 39217, USA; 6 Center for Computationally Assisted Science and Technology, North Dakota State University, NDSU Research Park DrP.O. Box 6050, Fargo, ND 58108, USA; 7 Blue Marble Space Institute of Science, 2800 Woodley Rd. NW #544, Washington, DC 20008, USA; 8 Earth-Life Science Institute, Tokyo Institute of Technology, 2-12-1-IE-1 OokayamaMeguro-ku, Tokyo, 152-8550, Japan; 9 Center for Chemical Evolution, Georgia Institute of Technology, North Ave NW, Atlanta, GA 30332, USA; 10 Institute for Advanced Study, 1 Einstein Drive, Princeton, NJ 08540, USA

## Abstract

Using novel advances in computational chemistry, we demonstrate that the set of 20 genetically encoded amino acids, used nearly universally to construct all coded terrestrial proteins, has been highly influenced by natural selection. We defined an adaptive set of amino acids as one whose members thoroughly cover relevant physico-chemical properties, or “chemistry space.” Using this metric, we compared the encoded amino acid alphabet to random sets of amino acids. These random sets were drawn from a computationally generated compound library containing 1913 alternative amino acids that lie within the molecular weight range of the encoded amino acids. Sets that cover chemistry space better than the genetically encoded alphabet are extremely rare and energetically costly. Further analysis of more adaptive sets reveals common features and anomalies, and we explore their implications for synthetic biology. We present these computations as evidence that the set of 20 amino acids found within the standard genetic code is the result of considerable natural selection. The amino acids used for constructing coded proteins may represent a largely global optimum, such that any aqueous biochemistry would use a very similar set.

Life on Earth has adapted to an impressively broad range of environments in large part by constructing diverse protein polymers using a set of just 20 genetically encoded amino acids. Multiple lines of evidence suggest that a wide range of amino acids, including many not used in biologically encoded proteins, were available from abiotic synthesis prior to the origin of life^1^,^2^,^3^,^4^. Even assuming life's usage of amino acids was biased by plausible prebiotic sources, of which a plethora exist, it appears that at least half of the genetically encoded amino acids arose as “inventions” of early living systems - novel chemical derivations of simpler counterparts during early metabolic evolution^5^. Combined, these two insights suggest that the set of amino acids incorporated into genetic coding represents only a small fraction of the wider pool of alternatives that might plausibly have been used^6^,^7^. The set of genetically encoded amino acids could therefore represent a fundamental adaptation, shaped by natural selection to give maximum fitness advantage. This model has proven to be consistent with predictions of evolutionary growth of the amino acid alphabet^8^, and simple statistical analysis reveals that the genetically encoded amino acids do indeed collectively exhibit unusual physical properties relative to random sets of amino acids^9^.

While previous studies have found strong support for the idea that the 20 genetically encoded amino acids exhibit non-random, adaptive properties as a set^9^,^10^, the strength of these findings is limited by the scope of alternative amino acids considered. Previous analyses considered a total of at most 76 amino acids: 50 that had been identified in the Murchison meteorite (representing prebiotically plausible amino acids including the coded amino acids Gly, Ala, Val, Pro, Glu, Asp, Leu, and Ile) the 12 remaining encoded amino acids not found in Murchison, and 14 intermediates of the metabolic pathways by which contemporary organisms synthesize amino acids (representing amino acids made available to organisms through evolutionary innovation). Recent work that applied chemoinformatics and structure generation to the question of the isomer space surrounding the genetically encoded amino acids indicates far more possibilities than previously imagined, numbering (depending on the structure generation criteria) in the range of several thousands to a few billion^11^. This finding calls into question the robustness of evidence regarding the adaptive qualities of the encoded amino acids relative to a background pool of only 76 alternatives. Are the perceived special qualities of the encoded amino acids simply an artifact of the comparison set's small size?

Here we test whether the observed non-random, adaptive properties for the set of genetically encoded amino acids remain robust when compared to a far larger and more comprehensive set of chemical possibilities than was previously available. We also begin to explore for the first time some “better sets,” which, given their adaptive qualities, might be plausible candidates for alternative biochemistries.

## Results

**Figure 1:**
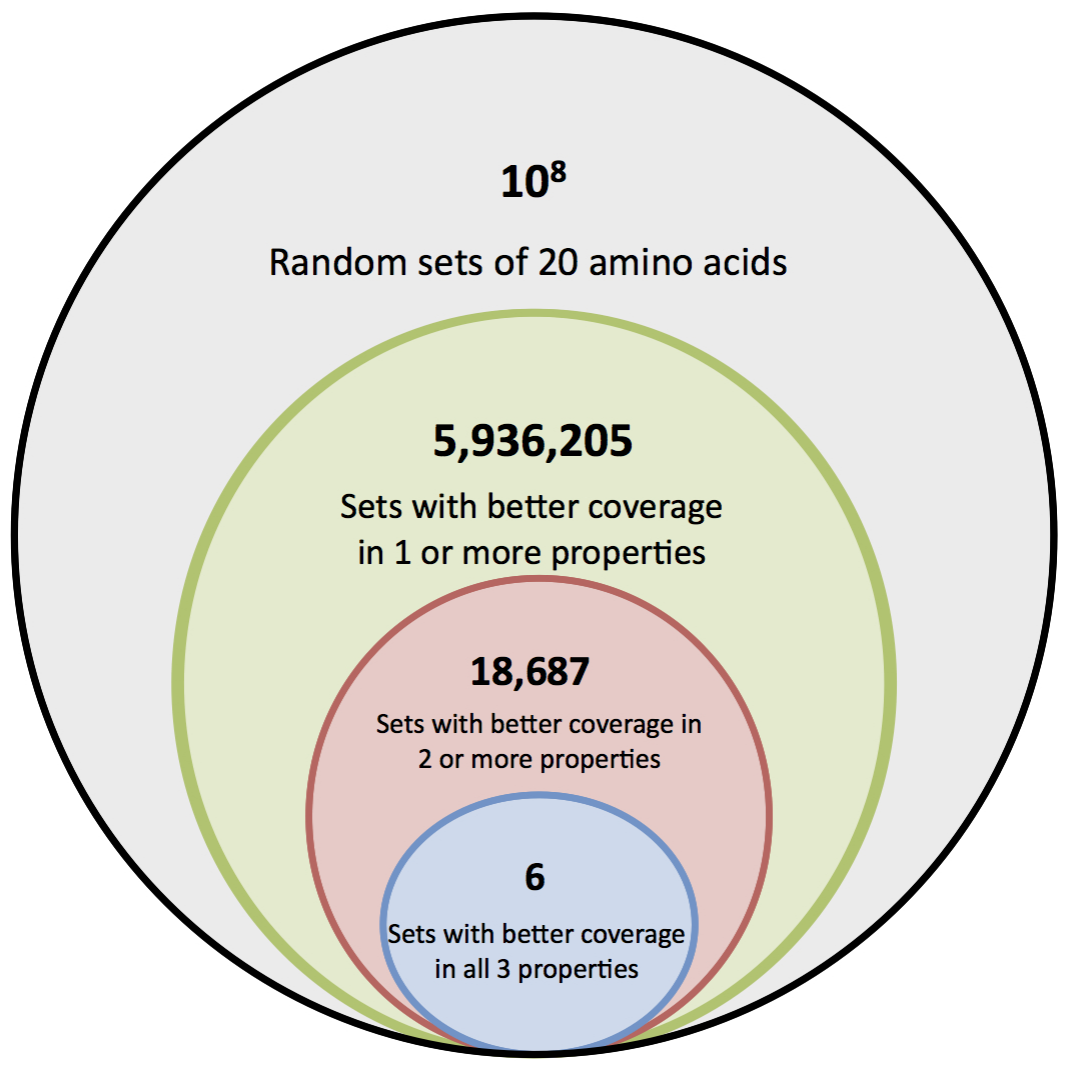
The number of random sets (out of 10^8^) with better coverage than the encoded amino acids in one, two, or three properties. Note that the circles are not drawn to scale; an appropriately scaled circle representing the number of random sets with better coverage in all three properties than the encoded set would only cover an area approximately 1/100^th^ of that of the period at the end of this sentence.

As described in the methods section, we drew 10^8^ random sets of 20 amino acids from our library of 1913 structures and compared their coverage of three chemical properties: size, charge, and hydrophobicity, to the standard amino acid alphabet. We measured how often the random sets demonstrated better coverage of chemistry space in one or more, two or more, or all three properties. In doing so, we found that better sets were extremely rare. In fact, when examining all three properties simultaneously, we detected only six sets with better coverage out of the 10^8^ possibilities tested. These results are summarized in Figure 1.

**Figure 2:**
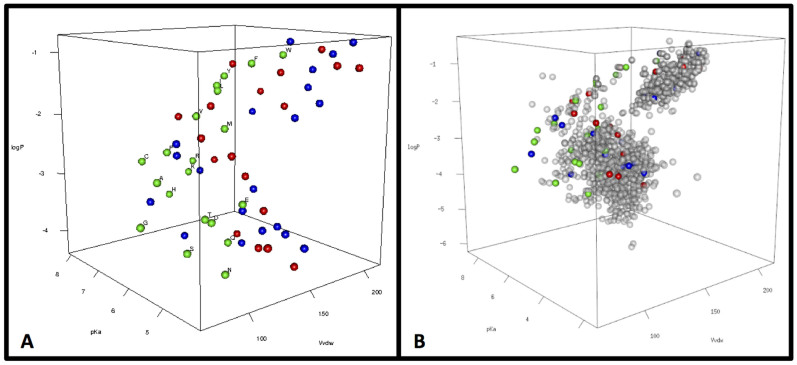
Figure 2a shows how amino acids occupy descriptor space. The genetically encoded amino acids (green spheres) are labeled by their one-letter abbreviations. Red and blue spheres represent two “better-coverage” (the first two sets from Figure 3). Figure 2b adds the remaining compounds of our virtual libraries as gray spheres. For animated and interactive representations see SI.

**Figure 3:**
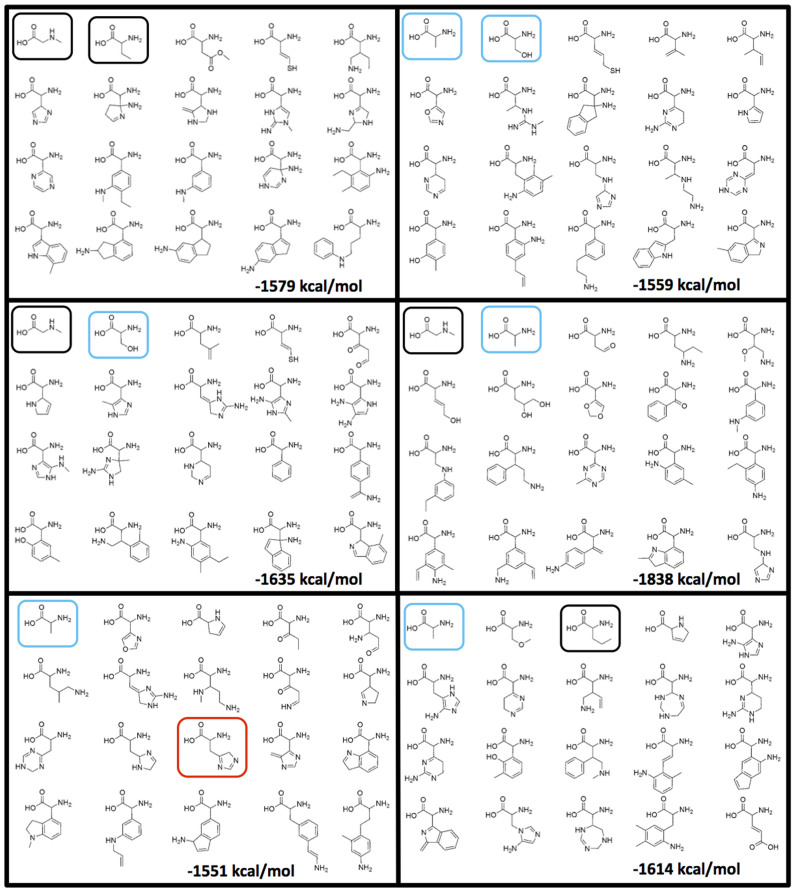
Six alternative amino acid sets detected within a sample of 10^8^ random sets that had better coverage than the encoded set of all three measured properties. The sum computed free enthalpy of formation of each set is provided. For reference, this value for the coded set is -2306 kcal/mol. Black cartouches represent amino acids identified in meteorites, light blue those found in both meteorites and the encoded set, red those found only in the encoded set.

We also programmed our search to save the identities of the molecules in the cases when better sets were found. Figure 2 shows three-dimensional plots of select better sets along with the encoded amino acids in the ‘chemistry space’ of size, charge and hydrophobicity, allowing visualization of property space coverage. While these three dimensions of property space are sufficient to demonstrate the adaptive advantage of the encoded amino acids, they are necessarily reductive and cannot capture all of the structural and energetic information contained in the “better coverage” sets. Figure 3 therefore illustrates the molecules comprising the six amino acid sets that exhibit better coverage than the encoded amino acids in all three physical properties, as well as their summed heats of formation (ΔH_f_°). When compared with the coded set, in no case was an alternative set also less costly in terms of total ΔH_f_° (ΔH_f_° more negative). In other words, the genetically encoded set of 20 amino acids once again meets the expectations of a hypothesis based on natural selection.

## Discussion

The results of the analysis of the 1913 chemical structures presented here corroborate and strengthen previous analyses that used a much smaller set of only 76 amino acid structures^9^. With only 6 · 10^−6^% of conceivable sets being as good or better, the encoded amino acids' coverage of chemistry space is remarkable regardless of the size of the background to which they are compared. This is consistent with the hypothesis that natural selection influenced the composition of the encoded amino acid alphabet, contributing one more clue to the much deeper and wider debate regarding the roles of chance versus predictability in the evolution of life (e.g. Ref. 12).

Even a library of several thousand molecules underestimates the true number of plausible amino acid chemical structures that might plausibly have entered into the genetic coding of living systems^11^. Our study is, however, the first to leverage the potential for computational chemistry to create and analyze densely populated chemistry space. The results of this analysis provide good reason to think that the highly unusual nature of the set of genetically encoded amino acids is not an artifact of the depth to which a background of possible amino acid structures is constructed.

While some of the molecules present in our library could be criticized for their likely instability, this does not create a bias with respect to the descriptor values. In other words, even if many molecules were culled from the library used here, the unusual nature of the coded set would persist.

Recording the sets of amino acid structures that appear to cover chemistry space as well or better than the encoded amino acids allows us to examine the properties of these better sets. Although six sets are a small sample of all possible better sets, interesting commonalities are observed. We first note that five of the six computed better sets (~83%) include one or more of the encoded amino acids. The probability that any given random set of 20 amino acids contains at least one genetically encoded amino acid is only 19%. Alanine and serine show up considerably more than would be expected by chance, whereas glycine never appears in any set (although sarcosine, a close structural homolog, appears three times). Perhaps not surprisingly, histidine, which also remarkably appears once, and serine lie on the outer edges of the 3-dimensional property space defined by the coded set (see Figure 2a). Interestingly, meteoritic amino acids are also highly represented in our better sets. 21 of the 37 meteoritic α-protonated-α-amino acids reported in carbonaceous chondrites^13^ overlap with our library. 14 of those 21 are encoded amino acids, seven are non-coded. The probability to have at least one of these non-coded meteoritic amino acids in a random set of 20 is only 7.1%. However, again, five of the six sets depicted in Figure 3 contain at least one non-coded meteoritic amino acid.

Functional criteria, such as the inclusion of certain functional groups, are not explicitly considered as dimensions of chemistry space in our analysis – indeed, the point of “chemistry space” is to abstract beyond these specifics^14^. Nonetheless, we observe interesting patterns in the distribution of functional groups within our better sets. For example, only three of the six sets include amino acid side-chains with sulfhydryl functional groups, and only one contains a carboxylic acid. Novel motifs that do occur include pyrrole (though the so-called 22^nd^ amino acid, pyrrolysine also includes this ring system) pyrazine, triazine, pyridine, pyrimidine, isoxazole, and isoindole ring systems, and aldehyde, ketone, ether and ester functional groups. Many of these also contain aromatic moieties substituted directly to the α-carbon atoms of the amino acid backbone, which may make these prone to epimerization – another example of how the encoded amino acids may be optimized beyond the simple considerations of our tests. Indeed, side chains of the better sets also include nucleophilic groups positioned flexibly at distances from the backbone that could facilitate peptide scission, a point noted as a possible explanation for the absence of homoserine, or homocysteine and its analogues, from the coded set^15^.

Ring containing structures are heavily represented in the total set (only 306 of 1913 (~16%) are acyclic), whereas the hypothetically more optimal sets contain an average of 29% acyclic amino acids, and the coded set contains 80% acyclic structures. In other words, optimal sets defined by our criteria are biased against the inclusion of ring-containing structures. The hypothetical better sets also contain a number of structures of potentially questionable hydro-, redox or photolytic stability. That none of the hypothetically better sets has a lower ΔH_f_° than the coded set strongly suggests that metabolic energetic concerns have been guiding forces in the natural selection of biology's set.

These observations combined suggest that additional factors beyond selection for the three properties principally considered in our test contributed to the adaptability of the coded set as a LUCA organism colonized habitable spaces on Earth. Given that each additional criterion greatly reduces the number of better sets, it would seem that adding functional criteria would only make the coded set even more unusual, and possibly reflect the truly limited set of possibilities that life has to choose from. What is remarkable within the analysis presented here is how few (and how simple) are the criteria required to perceive the encoded amino acids as a highly unusual set.

Many lines of evidence suggest that the amino acids were not recruited by biology all at once, but rather some may have been initially provided by environmental syntheses while others were added stepwise as novel biosynthetic pathways became available during evolution^5^,^8^,^16^,^17^,^18^,^19^. Thus, other questions could be asked about how the order in which amino acids are introduced affects the overall optimality of a set, or how changing the number of amino acids affects the optimality of sets that can be constructed. Given a mapping of three nucleotides to one amino acid, a highly redundant code could be constructed with fewer members that gives the same range and evenness of coverage, but that would lack the nuance of a larger, more diverse set. Ideas similar to this have been explored for “error minimizing” properties of the code^19^ but not for the concept of coverage of chemistry space.

Likewise, it should in principle be possible to compute plausible metabolisms which could connect the amino acids in the alternative sets, and it may be that metabolically "tighter" sets exist among them. This would form a useful target for future work.

The sets that exhibit better coverage of chemistry space than the genetically encoded amino acids appear to achieve this coverage in different ways. This raises the question: would an alternative biochemistry using such an amino acid alphabet have access to protein folds beyond the apparently finite repertoire known from terrestrial biochemistry? As *ab initio* folding software continues to improve, it could be used to explore this question. Quite aside from a relevance here for detecting alternative origins for life^20^,^21^, the recent demonstration of the stable incorporation of two new nucleotides into bacterial DNA has opened the possibility of adding hundreds of new coded amino acids to the artificial biochemical repertoire^22^. Analyses such as the one presented here might be useful for determining which amino acids could be added to such a "super-organism" so as to most extend its ability to explore novel protein space.

## Methods

### Generating background molecules

The starting point for our analysis was a previously computed set of α-amino acid structures^11^. Using molecular structure generation software based on principles of graph theory and constructive combinatorics^23^, this study computed two virtual amino acid libraries, a combined library (CL) including isomers of the 20 coded amino acids and their sub-formulas, and a unique library (UL), based on a unique fuzzy formula representing the complete formula range of the coded α-amino acids up to a certain number of C atoms. Due to the combinatorial nature of generating isomers and the concomitant exponential growth of the number of structures with increasing number of atoms, the latter library proved to be unwieldy, estimated to result in a library of more than 10^12^ structures^11^. We therefore chose to use the smaller library, which does not represent all possible amino acids in the size range of the encoded amino acids but gives far more comprehensive insight than previous studies into the chemical possibilities available to early life. We further filtered the 3,846-member library to exclude what were deemed to be especially unstable structures (mainly hemi-aminals). This left a final set of 1913 molecules that represent likely stable structural isomers of the 20 genetically encoded amino acids and their sub-formulas, including the biologically encoded 20 themselves. The set is available for download as an SD file, see SI.

### Definition of adaptive properties for a set of amino acids

In order to test the adaptive properties of the genetically encoded amino acids, we followed the hypothesis of Philip and Freeland^9^, that a well-formed set of amino acids should be distributed evenly across a broad range of values for key physical properties. In other words, a set of amino acids with *broad* and *even* distribution within a given property space would provide adaptive advantage during the evolutionary discovery of novel proteins. These two characteristics combined define the set's *coverage* of chemical property space.

### Choice and prediction of physico-chemical properties

Following the precedent of previous analysis^6^,^7^,^9^, as well as broad consensus on what defines functional properties of amino acids within proteins (reviewed in Ref. 7 and corroborated by recent meta-analysis^24^), we chose three chemical descriptors: size, hydrophobicity, and charge. This represents a carefully and thoughtfully selected alternative to considering any number of the thousands of available amino acid molecular descriptors in order to ask which, if any, cause the genetically encoded set to appear unusual. Our intention was to minimize the risk of introducing fallacious, *a posteriori* reasoning. That is, we wanted to avoid detecting the properties of genetically encoded amino acids that natural selection has “seized upon” over the course of billions of years of biological evolution and mistaking these for the properties that guided incorporation into genetic coding. Because the majority of the amino acids considered in our analysis are computationally generated molecules, we used chemical property prediction software to calculate quantitative values for our chosen descriptors. Accurate prediction of van der Waals volume (V_vdW_ - a measure of the total volume of the molecule enclosed by the van der Waals surface, for size) and log P (the partition coefficient, a measure of the distribution of the molecule between two solvents, typically 1-octanol and water, for hydrophobicity) is straightforward (see Ref. 25 for V_vdW_ and Ref. 26 for log P). These values were calculated using MOLGEN-QSPR^27^. Charge, however, is less easily predicted^6^. We chose to calculate pK_a_ rather than pI as a measurement for charge because there are fewer experimentally determined values of pI available with which to train prediction software. Our pK_a_ values were computed using ChemAxon's JChem package (http://www.chemaxon.com). To obtain values for an amino acid's side-chain in a polypeptide-like context, we first modified each amino acid by acetylating the α-amino group and converting the α-carboxyl group to an N-methylamide. As the library was initially generated using a single trivalent atom to substitute for the core H_2_NCH(C)COOH (for the sake of computational speed, see Ref. 11), the accurate CH_3_CONHCH(C)CONHCH_3_ substructure was reinstated prior to pK_a_ calculations using MOLGEN-COMB^28^. The pK_a_ values of all functional groups within the range from 2 to 14 in each molecule were then averaged.

### Calculations of heats of formation

The three physico-chemical properties of amino acids used in our tests are likely not the only characteristics that render amino acids adaptive, either individually or as a group. In addition, a functioning set of amino acids must be compatible with the organism's metabolism, and, all else being equal, natural selection would select a metabolism that is as efficient as possible^2^. For example, all of the coded 20 amino acids are interconnected by the network of metabolic reactions by which they are synthesized and decomposed. Presumably the same principle would hold any biochemistry^29^,^30^. Furthermore, the metabolic “cost” of an amino acid correlates with its usage in extant organisms in a manner consistent with natural selection for efficiency^31^,^32^,^33^. It would therefore seem likely that, all other things being equal, the most adaptive coded set would be one which allows for the greatest and most even exploration of descriptor space while at the same time being the least metabolically costly. In fact, it has already been noted that the encoded amino acids, particularly those thought to have been early additions to the genetic alphabet, have low thermodynamic “cost”^34^,^35^. To test this idea in the context of our study, we calculated one additional parameter – the enthalpy of formation (ΔH_f_°) of each molecule.

Calculations of heats of formation, ΔH_f_°, were performed by the RM1 method implemented in the semi-empirical quantum chemistry software package MOPAC2009 (MOPAC2009, J. J. P. Stewart, Stewart Computational Chemistry; Colorado Springs, CO, USA). Though sometimes perceived as less accurate than Density Functional Theory (DFT) methods, semi-empirical methods exhibit similar estimation error in the case of amino acids and are computed much more quickly. In particular, the RM1 method utilized here is a reparameterization of AM1, *i.e.* all RM1 parameters are optimized over those of AM1^36^. RM1 is therefore much more precise than the previously used AM1 and PM3 methods and has the same level of accuracy as PM6. As has been shown using a large set of molecules, the RM1 method is able to predict geometries and heats of formation consistent with DFT results and experimental observations^36^. The speed of MOPAC2009 and improved accuracy of RM1 are particularly valuable for generating electronic descriptors for structure–activity and structure-property relationship analyses. Recently, Puzyn et al.^37^ recommended the use of semi-empirical methods in QSAR/QSPR studies instead of the much more computationally-expensive DFT methods. The structures of amino acids were optimized to get conformations with minimal energy. The RM1 geometry optimizations of amino acids were carried out using the eigenvector following (EF) optimization procedure with a final gradient norm of the energy gradient less than 0.1 kcal/mol.

We chose not to consider ΔH_f_° as an additional dimension of chemistry space as it measures something fundamentally different, though no less important, from physico-chemical property descriptors. Our chosen three descriptors estimate the corresponding properties of possible polymers given a set of amino acids, whereas ΔH_f_° helps to constrain how easily these sets of molecules can be synthesized, be it abiotically or biochemically. In extant biological systems there is a strong correspondence between frequency of use of an amino acid in a protein and its biosynthetic cost^32^,^2^, however this is a measure of how frequently an amino acid is used to provide some aspect of chemistry space, not of how that space is occupied by that set.

### Selection of Random Sets and Comparison of Property Space Coverage

Using this unprecedentedly large pool of comparison α-amino acids and their calculated physical properties, we tested the encoded amino acids by measuring their coverage (*i.e.* how broadly and evenly they span chemical property space) for size, charge and hydrophobicity relative to sets of 20 amino acid molecules chosen from a much larger pool.

Each random set of 20 α-amino acids was drawn from the background pool without replacement, in order to determine whether it exhibited better coverage of any one, any two or all three physical properties. We repeated this calculation 10^8^ times in order to determine what percentage of random sets had better coverage than the encoded set. Since coverage is a proxy for the adaptive value of a given set of amino acids, our hypothesis (of an amino acid set selected for its adaptive properties) predicts that the encoded amino acids should exhibit better coverage of pertinent chemical property space than a significant portion of randomly selected amino acid sets.

## Author Contributions

M.I. analyzed data, wrote the manuscript, and produced all figures. M.M. advised the chemoinformatics workflow. S.F. was integral to project conception. B.R. performed quantum-mechanical calculations for the dataset. H.J.C. supervised the project and curated amino acid libraries. All authors reviewed and edited the manuscript.

## Supplementary Material

Supplementary Information Supplementary Information
