# Supplementary material for: Extraordinarily Adaptive Properties of the Genetically Encoded Amino Acids
Source: Sci Rep. 2015 Mar 24;5:9414. doi: 10.1038/srep09414 (PMC4371090; doi:10.1038/srep09414)
Supplement: Supplementary Information [file srep09414-s1.doc]

**Extraordinarily Adaptive Properties of the Genetically Encoded Amino Acids**

**Melissa Ilardo^a,b*^, Markus Meringer^c^, Stephen Freeland^d^, Bakhtiyor Rasulev^e,f^, H. James Cleaves II^g,h,I,j^**

*corresponding author

**Supplementary Information**

The compound library of 1913 amino acid structures as SD file can be downloaded from www.molgen.de/data/AACLBR.sdf.zip.

Animated GIFs of Figure 2 are available at

www.molgen.de/graphics/AdaptPropCodedAA/Fig2a.gif and www.molgen.de/graphics/AdaptPropCodedAA/Fig2b.gif,

Interactive WebGL representations are accessible via

www.molgen.de/graphics/AdaptPropCodedAA/Fig2a/index.html and www.molgen.de/graphics/AdaptPropCodedAA/Fig2b/index.html .
